# Feeding Strategies to Reduce Nutrient Losses and Improve the Sustainability of Growing Pigs

**DOI:** 10.3389/fvets.2021.742220

**Published:** 2021-10-28

**Authors:** Candido Pomar, Ines Andretta, Aline Remus

**Affiliations:** ^1^Sherbrooke Research and Development Centre, Agriculture and Agri-Food Canada, Sherbrooke, QC, Canada; ^2^Faculdade de Agronomia, Universidade Federal Do Rio Grande Do Sul, Porto Alegre, Brazil

**Keywords:** low protein diets, sustainable pig production, precision feeding, precision nutrition, nutrient utilization, nutrient efficiency of utilization

## Abstract

The efficiency of pig production using nutrients has increased over the years. Still, better efficiency of nutrient utilization can be achieved by feeding pigs with diets adjusted to their estimated requirements. An increase in nutrient efficiency of utilization represents economic gains while maximizing environmental performance. The objective of this paper is to review the impact of different methods of diet formulation that provide farm animals with the amount of nutrients to satisfy their needs while minimizing nutrient excretion and greenhouse gas emissions. Diet formulation is one tool that can help to maximize nitrogen and energy utilization by decreasing crude protein content in diets. The use of local feedstuff and non-human-edible products (e.g., canola meal) associated with synthetic amino acid inclusion in the diet are valuable techniques to reduce carbon footprint. Precision feeding and nutrition is another powerful tool that allows not only daily tailoring of diets for maximal nutrient efficiency of utilization but also to reduce costs and improve nitrogen efficiency of utilization. In this review, we simulated through mathematical models the nitrogen and energy efficiency of utilization resulting from crude protein reduction in the diet. An 8% crude protein reduction in the diet can increase nitrogen efficiency of utilization by 54% while costing 11% less than a control diet without synthetic amino acids. The same reduction in crude protein represented a major improvement in available energy due to the decrease of energetic losses linked to protein deamination. Urinary and hindgut fermentation energy losses were 24% lower for pigs fed with low-protein diets when compared to control diets. In terms of modern feeding techniques and strategies, precision feeding and nutrition can decrease nitrogen excretion by 30% when compared to group phase feeding. The benefits of feeding pigs with low-protein diets and precision feeding techniques are additive and might result in a 61% nitrogen efficiency of utilization. There is room for improvement in the way nutrient requirements are estimated in pigs. Improving the understanding of the variation of nutrient utilization among pigs can contribute to further environmental gains.

## Introduction

Farm animals are raised to produce commodities such as meat, dairy products, and fiber. Energy, amino acids (AA), minerals, vitamins, and water are used by animals for body maintenance, growth, reproduction, and lactation. Body maintenance and the synthesis of body tissues (i.e., lean, fat, etc.) are dependent upon an adequate supply of dietary nutrients (1). The energy and nutrient losses associated with the conversion of dietary energy and nutrients into animal products increase production costs and may also contribute to an environmental load of animal farms by the excessive application of nitrogen (N), phosphorus, or trace minerals from manure or by the carbon and methane losses. The conversion rate of dietary nutrients into animal products is generally low. Dietary crude protein (i.e., nitrogen), which is one of the most limiting and expensive nutrients in monogastric feeds, is converted to body protein by pigs with efficiencies that vary between 15 (2) and 33% (3). Similar figures are found for beef cattle and broilers, in which the efficiency ranges from 10 to 20% and from 30 to 40%, respectively (2). Nonetheless, given the global human population growth and the increasing demand for vegetable protein for human and livestock production, the method we are using to evaluate production efficiency needs to be redefined (4–6). For the efficiencies of conversion of human-edible livestock feeds into human-edible animal products, it may be more appropriate to evaluate these efficiencies in the actual context of limited global land resources and food security rather than just the efficiency of conversion of livestock feeds into units of animal products (4, 7, 8). For instance the use of digestible indispensable amino acids score to quantify differences in protein quality together with the concept of human-edible protein conversion efficiency allows to quantify the net protein contribution of a system (9, 10). Pig and chicken net protein contribution are around 0.64 and 0.76, respectively, while dairy cows will reach a 3.6 score (11). A score >1 indicates that the animal chain has a positive impact on providing human nutrients. Although these calculations are highly impacted by the feedstuff used in pig and poultry diet, the numbers are pointing for a competition for food between humans and non-ruminants. The challenge to animal scientists and the livestock sector is to improve the efficiency of use of feed resources by matching available nutrients to the animal requirements while reducing the livestock dependence on human-edible feeds (6, 7, 12, 13).

The efficiency by which farm animals convert the dietary nutrient provisions into animal products depends on many factors. These factors can be associated with the animal (i.e., its metabolism, age, and species), the feeding method (i.e., feed composition, feeding phases), and the environment (i.e., housing system). Within the animal, there are various causes of nutrient inefficiency. Thus, part of the ingested nutrients are used for basal metabolic processes involving degradation (catabolism) and synthesis (anabolism) or are lost in the digestive tract through desquamation and endogenous secretions (14). These nutrient losses are generally referred to as maintenance losses. Nutrients are also lost during the synthesis of animal products (e.g., body lean). In growing animals, the losses associated with the utilization of the first-limiting AA for body protein deposition can largely be attributed to its inevitable catabolism (14, 15). These inevitable AA losses should be differentiated from other metabolic losses related to the preferential AA catabolism, which results from the catabolism of AA given in excess, from the excretion of chemically unavailable absorbed AA (e.g., heat-damaged proteins) (16, 17), and to a minor extent from integumental AA losses and from the use of AA for the synthesis of non-protein body compounds (14). In growing pigs fed with cereal-based diets, the sum of the undigested N and the losses associated with digestion, maintenance functions, and body protein deposition may represent 33% of the total ingested N, and similar values are obtained for dietary P (3). These sources of nutrient inefficiency are difficult to reduce because they are inherent to the animal metabolism and occur during digestion and metabolic processes (18).

Other sources of nutrient losses are related to the composition of the feeds and the methods we use to provide these feeds to the animals. Because these losses are related to the way we are feeding and raising the animals, there is great potential for improvement. Indeed, the feeds are responsible for the largest part (70%) of the environmental impact caused by pig production (19, 20). This is because in practical conditions most of the pigs within the herd receive more nutrients than they need (21–23), and all excess nutrients are excreted and contribute to the overall nutrient inefficiency. To reduce the supply of excess nutrients and thus reduce their excretion, it is essential to: (a) precisely estimate the amount of dietary nutrients that will be available for the animals' metabolism; (b) estimate the amount of nutrients required by each animal throughout the growing period; (c) formulate balanced diets that limit excess nutrients; and (d) concomitantly adjust the dietary supply of nutrients to match the animals' estimated requirements (24). The estimation of available nutrients in the available feed ingredients and the determination of nutrient requirements have been previously addressed (25, 26). Additionally, the environmental impact of livestock production must also include the direct and indirect contribution of farm animals and manure disposal to greenhouse gas (GHG) emissions, which in some cases, like in pig and poultry production, may contribute to around 9.5% of the global livestock GHG emissions (27, 28). The objective of this paper is to review the impact of the different methods of diet formulation to provide growing pigs with the amount of nutrients that satisfy their needs and concomitantly minimize their excretion and GHG emissions.

## Formulating Balanced Diets to Reduce Nutrient Losses and Excretion

Formulating a compound feed for farm animals refers to the determination of a blend of feed ingredients and additives that will have the concentration of nutrients that will allow the achievement of the production goals at an optimized feed cost (29). A compound feed is said to be complete when it provides all the nutrients required by animals. Many farm animals are fed today with complete diets.

One of the essential requisites for diet formulation is to precisely know the nutrients in feed ingredients that will be available to the animals after digestion and the amount of nutrients that are required by the animal to live and produce. Linear programming is the most widely used method for diet formulation and involves determining the level of incorporation of the available feed ingredients that, by respecting a series of linear constraints, will minimize (or maximize) an objective function, typically the cost of the blend. Other methods, such as goal programming, are proposed as an extension of linear programming to include several optimization criteria (30). Nonetheless, the main characteristics of these methods are the result of the linear nature of the objective function and constraints (31), which requires the verification of important assumptions such as the additivity (the value of the objective function is the sum of the contributions of each ingredient, and, similarly, the nutritional contribution of a blend of ingredients is the sum of the nutrient contribution of each ingredient), proportionality (the change in the contribution of an ingredient in a blend changes the nutritional value and cost of the blend in proportion to the change) and divisibility (the incorporation of an ingredient in a mixture is divisible indefinitely, and there are no ingredient or nutrient interactions).

For any nutrient, feed ingredient provisions and animal requirements can be expressed in different units or within different nutritional systems. The system and units used to appraise the potential nutrient contribution of feed ingredients and those required by animals have to verify these assumptions of the formulation method. For example, the apparent ileal digestibility of AA does not satisfy the additivity assumption, because animal responses to increasing levels of an AA are not necessarily linear (32). The use of net energy and standardized ileal digestible AA systems circumvent these limitations (32–34).

Furthermore, AA requirements are often expressed based on the concept of the ideal protein. The ideal protein concept was proposed more than 50 years ago and refers to a protein in which all dietary essential AA and the pool of dietary non-essential AA are co-limiting so that AA supply exactly matches the AA requirement (35, 36). Lysine has traditionally been used as the reference AA because it is the first limiting AA when pigs and poultry are fed with corn-soybean meal based feeds. The utilization of the ideal protein concept greatly simplifies practical animal nutrition and feed formulation, because the nutritionist only has to evaluate the requirement of lysine and extend the requirements of the other AA using the ideal protein profile.

Nonetheless, the scope of the conventional diet formulation methods is to satisfy the nutritional constraints while minimizing the cost of the blended feed and the supply of excess nutrients when adding environmental constraints. Other than the limitations inherent to the linearity of the objective function and constraints, and the assumptions identified above, linear programming is limited by the objective function, which is normally proposed to minimize the cost of the feed (i.e., the blend). In other words, what counts is to provide the necessary nutrients independently of their origin. Thus, two diets are assumed to be equivalent if they satisfy all the nutritional constraints of the formulation method independently of the nutrient excesses they provide. Unfortunately, reducing the environmental footprint by adding environmental objectives in the diet formulation method is often considered a complex and costly task that adversely affects production competitiveness. Introducing environmental objectives in the diet formulation algorithms can be accomplished by modifying the traditional least-cost formulation algorithm (37–39), using goal and other programming techniques (30, 40–42) and others. However, whatever formulation method is chosen, the environmental criteria to be minimized must be those that will have the greatest impact on the environmental footprint of production. The use of life cycle assessment to globally quantify this environmental footprint is a promising avenue (43) but has the downside that it attributes to the livestock feed the environmental footprint associated with the production of ingredients, fertilizers, etc. The resulting solution may be optimal for society in general, but it will not necessarily be optimal for the production sector or the producer himself. The practical use of this approach will require the adoption of national and international policies allowing the sharing of the environmental costs between consumers and the various stakeholders in the sector (4). Only the environmental footprint associated with animal feeding is considered in this study.

### Mitigate the Carbon Footprint by Feed Formulation

With the increasing demand from society to reduce the global environmental carbon footprint of animal production systems with a focus on improving the sustainability of the production of feed ingredients, the utilization of these ingredients by livestock and the disposal of manure is warranted. Thus, other than formulating the feeds to reduce nutrient losses and excretion, more strategies are required to mitigate global production carbon footprint. Thus, (1) formulating feeds using local ingredients, (2) using by-products from the food and bio-energy industry, (3) formulating low-protein diets by increasing the use of crystalline AA, and (4) using more efficient crops with reduced fertilizer (e.g., precision farming) have been proposed (44). Between all these strategies, the use of more efficient crops can help to decrease the carbon footprint. However, when considering changes in land use, low-protein diets with crystalline AA seems to be the most efficient strategy to mitigate the carbon footprint (44). Crystalline AA are synthetically made but present with the same configuration as naturally occurring AA. The use of feed-grade AA allows replacing bound protein by synthetic and crystalline AA (45, 46). Amino acids can be produced by the different methods such as: extraction from protein hydrolysates, chemical synthesis, and microbial processes; each method presenting different economic and environmental advantages (45, 47). Crystalline AA are the product of bacterial fermentation which is purified by crystallization (45). In production contexts like in Europe, where feed ingredients are frequently imported from distant countries like Brazil and Argentina, reducing the utilization of soybean meal by using feed-grade AA significantly decreases land use, carbon footprint, and GHG emissions (43, 48, 49). Reducing soybean meal utilization can be attained by formulating low-protein diets by incorporating crystalline AA, by using precision feeding, or both. Nonetheless, these feeding alternatives are environmentally viable only if they do not compromise growth performance (50, 51).

### Simulated Impact of Low-Protein Diets in Nutrient Efficiency, Nutrient Excretion, and Carbon Footprint

Energy, AA, minerals, vitamins, and water are essential nutrients needed by animals to live (maintenance), grow, and produce (reproduction, lactation, etc.). When formulating a diet, it is necessary to consider that animals must be provided with all these essential nutrients in adequate amounts and in forms that are palatable, digestible, and metabolically available in order to optimize growth, reproduction and production (1). It is also assumed that for many nutrients, and particularly for AA, their excess will not compromise performance. In fact, the excreted N originates from the undigested, unbalanced, and chemically unavailable dietary protein fractions, from the protein given in excess to the animals, and from the inevitable protein catabolism (14). With the increasing availability of crystalline AA such as L-lysine, DL-methionine (or its analogs), L-threonine, L-tryptophane, and L-valine, it is now possible to formulate low-protein diets with a well-balanced AA content. When providing pigs and other monogastric animals with the required amount of essential AA, including the pool of non-essential AA does not affect animals' growth (1, 52–54).

The impact of low-protein diets in nutrient efficiency, nutrient excretion, and carbon footprint was evaluated by simulation feeding growing pigs with five feeds formulated to lower dietary crude protein (CP) content with the inclusion of different crystalline AA based on studies addressing the use of low-protein diets (44, 55). The feeds were formulated to meet the requirements of 25–50-kg body weight growing pigs (23, 56, 57) using the nutritional matrix of the NRC (1) for feed ingredients, the standardized ileal digestibility values of EvaPig® software (v. 1.4.0.1; INRA, Saint-Gilles, France), and recognized ideal protein AA profile (54, 58). The feed ingredients used to formulate the basal diet (Diet 1) contained corn, wheat, soybean meal, canola meal, vegetable oil, mineral sources (micro-mineral premix, calcium carbonate, dicalcium phosphate hydrated), and phytase. These feed ingredients were chosen from local sources when possible, while costs were those of January 2021 expressed in US dollars using the conversion rate of January 15, 2021.

Pig performance was simulated (59) based on previous study results (18, 58, 60), assuming that during a 28-d feeding phase starting at 25 kg BW, pigs will have an average daily feed intake (ADFI) of 2 kg, average daily weight gain (ADG) of 0.95 kg, and an average protein deposition (PD) of 152 g/d. Daily lysine requirements (g/d) were calculated by adding maintenance and growth requirements as generally suggested in the literature (53, 56, 57). Fecal energy losses were estimated by the difference between the gross and digestible dietary energy in diets. Urinary energy losses were calculated as suggested by van Milgen et al. (53), assuming that they originated from the deamination of two nitrogenous component fractions, one obligatory and another variable. The obligatory energy loss fraction is associated with maintenance, while the variable urinary energy excretion fraction is proportional to the excess protein supply. The difference between digestible and metabolizable energy represents the methane loss from fiber fermentation. Heat losses were obtained by determining the difference between the metabolizable and net dietary energy. These values were multiplied by ADFI to estimate average energy losses (MJ/day). Nitrogen and other nutrient excretion values were obtained by subtracting the estimated retention from the respective nutrient intake values.

**Figure 1:**
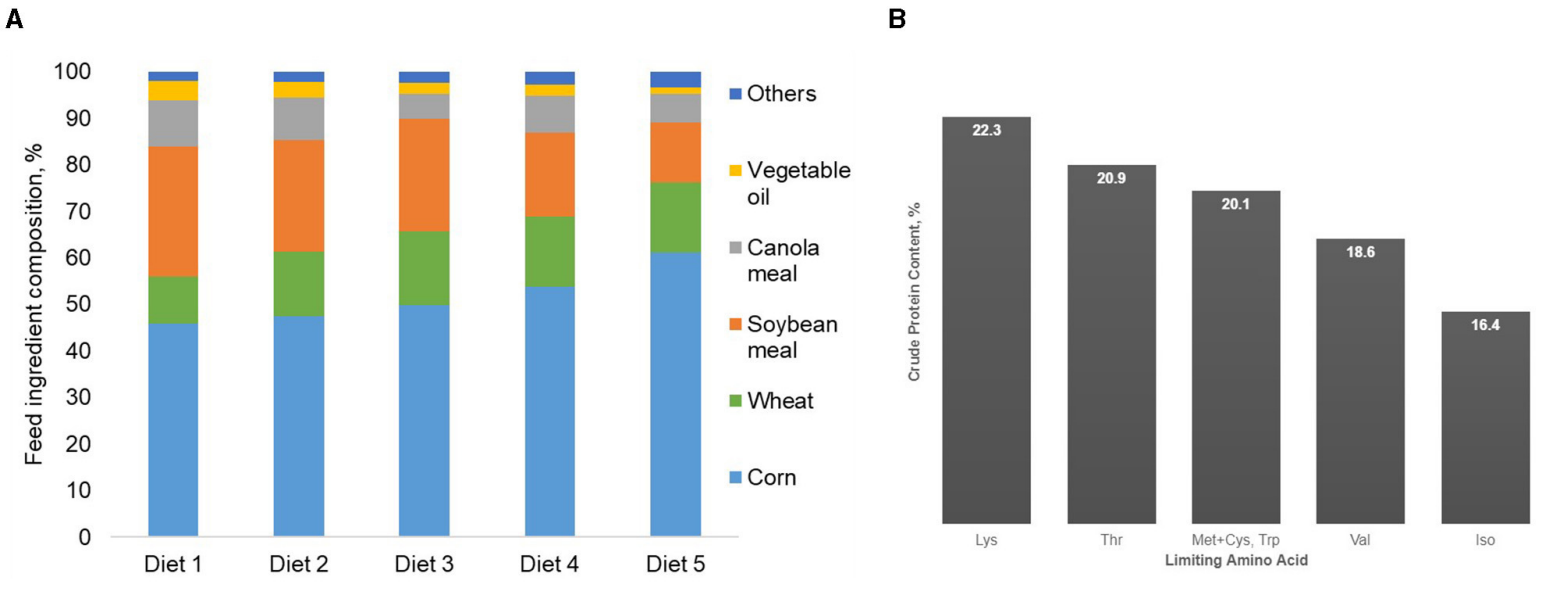
Feed ingredient composition of diets formulated for grower pigs (25–50 kg BW) in standard Canadian pig diets when soybean and canola meals are gradually replaced by corn and feed-grade amino acids **(A)**. Crude protein content and limiting AA of diets formulated for grower pigs (25–50 kg BW) in standard Canadian conditions when soybean and canola meals are gradually replaced by corn and feed-grade amino acids **(B)**.

In relation to growing pig AA requirements, corn is poor in lysine (1), which is generally the first limiting AA in the diets of many growing animals, including pigs. Because of this limited lysine content in corn, a higher amount of soybean meal has to be included in conventional corn-soybean meal diets to meet the lysine requirement of pigs, which results in high CP levels (55). The basal diet (Diet 1) formulated to satisfy the AA requirements of pigs without the addition of crystalline AA was mainly composed of soybean and canola meal, whose inclusion accounted for 38% of the diet and resulted in 22.3% CP diet (Figure 1A, Table 1). Supplementing this basal diet with L-lysine until the second essential AA becomes limiting (i.e., threonine; Diet 2) reduced dietary CP by 7% (Figure 1B). This decrease in dietary CP resulted from a decrease in soybean and canola meals and an increase in corn and wheat. In relation to the basal diet, a reduction of 10% in dietary CP can be obtained by supplementing the basal diet with L-lysine and L-threonine until the third AA becomes limiting (Diet 3). At this point, tryptophane and methionine became limiting, and by supplementing with these four feed-grade AA, a 17% CP reduction (19% CP content) can be obtained (Diet 4). Valine becomes the next limiting AA. Supplementing the basal diet with L-lysine, L-tryptophan, L-threonine, MHA-methionine, and L-valine resulted in a 26% reduction in the CP in Diet 5 (16% CP content). It is important to stress that the order of the limiting AA and the potential CP reduction in the diet depends on the nutritional matrix used, the ideal AA profile chosen, the economical scenario, and the estimated AA requirements of the animal.

**Table 1:** Estimated nutrient composition and simulated results of the diets formulated for grower pigs (25–50 kg body weight) in standard Canadian diets when soybean and canola meals are gradually replaced by corn and feed-grade amino acids.

	**Diet 1**	**Diet 2**	**Diet 3**	**Diet 4**	**Diet 5**
**Estimated energy and nutrient composition**					
Dry matter	87.74	87.63	87.46	87.45	87.26
Ash	4.96	4.82	4.64	4.66	4.39
Crude protein	22.34	20.88	20.11	18.64	16.48
Crude fat	6.63	5.96	5.07	5.07	4.29
NDF	9.8	9.81	9.42	9.78	9.58
ADF	4.7	4.52	4.1	4.27	3.9
Starch	35.8	38.96	41.43	43.4	47.77
Gross Energy, MJ/kg diet	17.23	16.99	16.74	16.65	16.36
Digestible Energy, MJ/kg diet	14.87	14.69	14.57	14.44	14.25
Metabolizable Energy, MJ/kg diet	14.15	14.01	13.91	13.82	13.7
Net Energy, MJ/kg diet	10.39	10.38	10.35	10.38	10.43
Total Lys, %/kg diet	1.19	1.17	1.16	1.15	1.13
SID^*^ Lys, %/kg diet	1.02	1.02	1.02	1.02	1.02
**Simulated animal responses^a^**					
Fecal energy losses, MJ/d	4.72	4.6	4.34	4.42	4.22
Urinary energy losses, MJ/d	1.44	1.36	1.32	1.24	1.10
Heat increment^c^, MJ/d	7.52	7.26	7.12	6.88	6.54
Nitrogen intake, g/d	69.70	65.15	62.74	58.16	51.29
Nitrogen retained as protein^b^, g/d	24	24	24	24	24
Nitrogen for maintenance^b,c^, g/d	3.4	3.4	3.4	3.4	3.4
Nitrogen excreted, g/d	42.26	37.71	35.30	30.72	23.85
Nitrogen retention, %	39.37	42.12	43.73	47.18	53.50

* *Standardized ileal digestible Lysine (Lys)*.

a *Assuming an ADFI of 1.95 kg/d, an ADG of 0.95 kg/d, and that 16% of ADG is deposited as protein*.

b *Hauschild et al. (56)*.

c *Van Milgen et al. (53)*.

**Figure 2:**
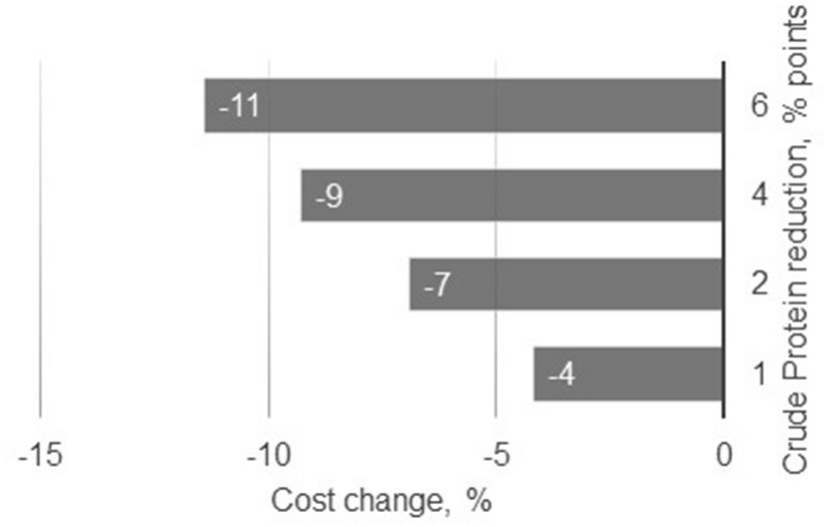
Changes in estimated feeding costs as result of changes in crude protein content in the diet.

The use of five feed-grade crystalline AA allowed a decrease in soybean and canola meals by 50% in relation to the basal diet (Diet 1). These feed ingredients accounted for 38% of this reference diet. Such reductions in protein-providing feed ingredients in livestock diets not only significantly reduces N excretion, but also contributes to reductions in land use and carbon footprint (48, 49). Nitrogen excretion was reduced in the present study by 8% per percent unit reduction in dietary CP, which is in agreement with Wang et al. (55), who reported reductions of N excretion of 8–10% for each percent unit reduction in dietary CP. In the present simulation study, the efficiency of N retention increased from 40 to 54% when pigs were fed with diets 1–5, respectively. Concomitantly, reducing dietary CP also reduced feed cost (Figure 2). Although feed cost continuously changes over time and across production contexts, Diet 5 was 11% cheaper than the control diet, resulting from the reduction of soybean and canola meal inclusion in the AA-supplemented diets.

Dietary gross energy is not totally available for meeting the requirements of animals, since some energy is lost in feces, in urine, as fermentation gases (methane, hydrogen) and as heat (i.e., heat increment). The energy losses that are found in the feces come from the organic matter of the diet that has not been digested by the animal (61). Fecal energy losses may represent 14% of the gross energy intake, while urinary and fermentation losses may represent 8% in non-supplemented diets. Feeding pigs with low-protein diets will reduce fecal losses by 11% given the higher energy digestibility of energy in cereals than in soybean and canola meals. Furthermore, low-protein diets will significantly decrease protein deamination, which was therefore the energy loss component that presented the greatest difference in energetic cost. Urinary and hindgut fermentation energy losses were 24% greater for pigs fed with the basal diet (22% CP) than with low-CP diet (16% CP), likely because 31.1 kJ of energy is needed to deaminate and excrete each g of excess N in the urine (1). Heat increment decreased by 13% between diets 5 and 1. Such a change in heat increment is mainly due to the change in the proportion of starch and protein content in the diet. Given that glucose is used more efficiently than protein as an ATP source (62), reducing excess protein also decreases heat increment. Furthermore, high dietary CP content stimulates body protein turnover, a process which increases energy expenditure (63).

## Precision Feeding as a Tool to Improve Nutrient Efficiency of Utilization

Reducing the excretion of excess nutrients and restricting the use of non-renewable resources are essential components in the development of sustainable livestock production systems. The amount of nutrients that are excreted depends mainly on how much nutrients are ingested, how metabolically available they are, and how their supply by the diet is balanced with the animals' requirements. In growing animals, the optimal concentration of nutrients in the diet progressively decreases over time (1). Therefore, an efficient way to reduce the excretion of excess nutrients is to concomitantly adjust their supply to the animals' requirements (64, 65). The economic and environmental benefits of this concomitant nutrient adjustment increase with the number of feeding phases (64, 66, 67). However, increasing the number of feeding phases complicates feed management and sometimes increases facility costs. The development of feeding systems that allow blend feeding and the automatic distribution of two feeds that, when combined in variable ratios, can meet the requirements of pigs throughout their growing period (64, 68) makes the phase-feeding technique promising again because nutrient excretion can be significantly reduced without increasing feeding costs (69). Nonetheless, there are two important sources of variation to be controlled in farm animals, which are the between-animal variation and the overtime variation on nutrient requirements (70, 71). Conventional farm animals are fed with the same feed during long periods (1, 72). Therefore, only the overtime variation can be controlled by increasing the number of feeding phases. Furthermore, given that for most nutrients underfed animals will exhibit reduced performance, whereas the overfed ones exhibit near-optimal performance, nutrients are provided to satisfy the requirements of the most demanding animals in the herd to ensure optimal production performance (i.e., growth) (21, 22, 73). In this situation, almost all animals receive more nutrients than they need. Furthermore, to account for the lack of information to precisely estimate the optimal level of nutrients to be provided to the group, the composition of feed ingredients, and other uncontrolled and unknown factors (e.g., environment, health), nutritionists include safety margins when formulating diets for maximum population responses.

Precision feeding or precision animal nutrition is the practice of feeding animals with diets tailored daily (71). Precision feeding and nutrition is part of the precision livestock farming approach and involves the use of feeding techniques that allow the proper amount of feed with the suitable composition to be supplied in a timely manner to individual animals or groups of animals (24, 74). The automatic collection of data by the use of interconnected smart sensors and devices and the use of big data analysis techniques combined with conventional mathematical and data-driven models using deep learning algorithms and control devices (i.e., automatic feeders) are required for precision feeding applications (71). The application of precision feeding at the individual level is only possible where measurements, data processing, and control actions are taken at the individual animal level (71).

The use of real-time feed-intake and body-weight information allows estimating the required amount of nutrients that a group of pigs (22) or each pig in the herd (56) needs to grow at its potential. For example, a real-time modeling-control approach was used by Pomar et al. (64) to control the time-dependent variation of group-housed pigs offered feed *ad libitum*. In this system only two feeds are used throughout the grow-finishing period: feed A, which has high nutritional density, and feed B, which has a low nutritional density (24, 74). The daily tailored diet is obtained by mixing the right proportion of these two feeds to each individual (individual precision feeding) or for a group of animals [daily-phase group-feeding system; (75)]. Comparing a conventional three-phase feeding system to a daily-phase group-feeding system, these authors concluded that CP intake could be reduced by 7% while N excretion is reduced by 12%. Controlling the time-dependent and between-animal variation can further help to reduce nutrient intake and excretion. The modeling approach proposed by Hauschild et al. (56) was used to estimate real-time nutrient requirements of individual pigs. The performance of growing pigs fed according to a conventional three-phase feeding system, similar to the one used by Pomar et al. (64), or using precision feeding were compared by Andretta et al. (75, 76) and Remus et al. (77), who observed that feeding pigs with diets in which the concentration of standardized ileal digestibility (SID) lysine is adjusted daily to the estimated requirements of each animal resulted in a 27% reduction in total lysine supply without detrimental effects on growth. This additional 20% reduction in SID lysine intake in relation to group-fed pigs was obtained by feeding the animals individually and thus simultaneously controlling the time-dependent and between-animal variation. Although feed cost reduction depends to a great extent on feed prices, it is expected that feed cost can be reduced by 1–3% when only controlling the time-dependent variation, while an 8–10% reduction can be obtained when controlling both sources of variation. Nitrogen excretion was reduced by nearly 30% when pigs were fed with daily tailored diets. The efficiency with which dietary protein was used for body protein retention was improved by 12.5% (75) and 13.4 % (76). Moreover, crude and SID lysine were improved in these trials by 30 and 23%, respectively. These differences between the CP and lysine efficiencies resulted from the fact that the experimental diets were not formulated to minimize CP content and the lysine to CP contents were different between feeds A and B.

### Formulating Low-Protein Diets for Precision Feeding

The benefits of feeding pigs with low-protein diets and precision feeding techniques are additive. Therefore, formulating diets for precision-fed pigs with crystalline feed-grade AA can dramatically reduce the carbon footprint of growing-finishing pig production. Thus, if the diets in the trial of Andretta et al. (76) would have been formulated as proposed for Diet 5 in the previous simulation exercise, we could theoretically expect reductions in N excretion up to 43% instead of the observed 26%, with an N efficiency moving from 54 to 61%. It is important to see from these trials that young animals are much more efficient than older ones and that feeding pigs under requirements dramatically improves N and other nutrients efficiencies. Indeed, feeding pigs at 90% of the estimated SID Lys requirements would decrease protein retention by about 5%, while N excretion can be reduced by nearly 20% in relation to pigs fed to requirements. This reduction is, however, very sensitive to the formulation method. In fact, the feeds formulated for young animals are more concentrated in all nutrients, including AA. Therefore, the use of feed-grade crystalline AA will have a greater effect on the reduction of total protein than feeds formulated for heavier animals. When the diets are formulated for precision feeding, again, the more concentrated feed responds more to the incorporation of AA than the less concentrated feed. On the other hand, the less concentrated feed (i.e., feed B) is normally formulated for the least demanding animals when they are the least demanding. Therefore, at the end of the growing period, the required levels of AA and other nutrients are low and they are less affected by the incorporation of crystalline AA. These less concentrated feeds do not require the incorporation of any protein-providing ingredient, given that even the AA concentration of cereals exceeds the required level for this feed.

### The Limitations of Actual Methods to Formulate Low-Protein Diets in the Context of Conventional and Precision Feeding Systems

The formulation of low-protein diets can have a great impact on livestock sustainability, but it is in using low-protein diets in precision feeding settings where the impact can be greater, given the additivity of both feeding techniques. Feeds and feeding remain the most important production factors to reduce the carbon footprint given that they account for around 70% of the environmental impact of pig and poultry production (20). Nonetheless, despite the tangible benefits of using low-protein diets and feeding pigs with efficient precision feeding systems, there are limitations to the actual principles we are using to formulate low-protein diets (23, 55) and for precision feeding systems (71).

Precisely adjusting the supply of nutrients to the needs of animals is the key issue to optimize the efficiency of use of feed nutrients and minimize their excretion and the environmental footprint of animal production systems. In the practice, the digestible AA content in the complete diet is obtained, assuming that the digestibility values of the feed ingredients are additive and independent of the animal, feed intake, and ingredient composition (32, 78, 79). However, these principles are weak, as low levels of feed intake may increase the estimated values of apparent ideal digestibility and SID of CP and AA in diets (78) and the inclusion of dietary insoluble fiber decreases the digestibility of most dietary components, including AA (80, 81). These phenomena may lead to the lack of additivity and the under- or overestimation of the available AA in the complete diet (78). Our ability to precisely estimate the available nutrients in feed ingredients and the final diet remains an important limitation to formulating low-protein diets or providing pigs with the amount of nutrients animals need for production.

On the other hand, the determination of the amount of nutrients that the animals need to produce may also be challenging. For specific nutrients (e.g., essential AA), and when all other nutrients are provided at adequate levels, nutrient requirements can be defined as the amount of nutrients needed for specified production purposes, which in farm animals are production outputs such as growth rate, protein deposition, and milk yield (82, 83). Depending on the production purpose and the nutrient, this required nutrient amount can be considered as the minimum amount that will prevent signs of deficiency and allow the animal to perform its necessary functions in a normal manner. Nutrient requirements are modulated by factors that are related to the animal (e.g., genetic potential, age, weight, and sex), the feed (e.g., anti-nutritional factors), and the environment (e.g., temperature and space allowance) (84) and they are estimated for a given animal at a given point in time as the sum of the requirements for maintenance and production (26). When applied to pig populations, however, the requirements for a nutrient should rather be defined as the amount needed for specified production purposes such as optimal growth rate, protein deposition and feed efficiency (22). That is, the concept of nutrient requirements when applied to populations should be considered in the context of nutrients provided to heterogeneous populations over long periods (73, 85, 86). Individual animals' response to dietary nutrient supply may differ in magnitude and pattern from the response of a population (73), and population nutrient requirements should be seen as the optimal balance between the proportion of pigs that are going to be overfed and underfed, acknowledging that this proportion will change over time (25).

The empirical and factorial methods are two methods used in practice to estimate the nutrient requirements of growing animals (29). In the empirical method, nutrient requirements are estimated by feeding groups of pigs with increasing levels of the nutrient under evaluation and measuring one or several sets of performance parameters (e.g., growth rate). In this empirical method, the nutrient level at which the optimal population response is observed is identified as the population requirement for this nutrient and this growing interval. In the factorial method, however, daily requirements are estimated as the sum of the requirements for maintenance and production (82). These requirements are estimated for each nutrient or its precursor and take into account the efficiency with which each nutrient is used for each metabolic function (53, 87). Because pigs within a population differ in terms of BW and growth potential, each pig has its requirement, and this requirement evolves over time according to each pig's own pattern of feed intake and growth. When the factorial method is used to estimate the nutrient requirements of a population of animals, it is common practice to use the average pig to represent the population. However, care has to be taken with this assumption, since using the average pig to feed the population implies that half of the population will be overfed while the other half will be underfed (21, 22, 26), thus leading to undesired population performance. Nonetheless, some factorial methods may have been calibrated to estimate the requirements of the population using average population values (23). Furthermore, unlike the empirical method, the factorial method estimates nutritional requirements using information from one individual at one specific point in time. Thus, changes that occur during the growing interval under study are not evaluated. Ultimately, both methods of estimating nutrient requirements are based on experimental results from trials studying the relationship between nutrient intakes and animal responses. In the empirical method, this relationship is used to estimate the optimal response to varying nutrient levels of a population of animals showing some degree of heterogeneity. In contrast, the factorial method estimates the required amount of nutrients for one animal at a given point in time. Thus, when the factorial method is used to estimate population nutrient requirements, the chosen individual should be the right representative of the population and not necessarily the average animal (22, 23, 73).

Mechanistic mathematical models that implement the factorial approach are used to represent the complexity of animal responses and the numerous factors modulating them. These models have been developed to simulate the growth of a single animal (1, 53) or a population (86, 88). These models must, however, be calibrated a priori using data collected from bygone reference populations. Furthermore, these models are challenged by the difficulty of identifying the right reference population for its calibration, the inadequacy of most of these models to represent population heterogeneity, and the fact that animals from actual populations may follow different feed intake and growth patterns than the ones observed in the reference population. Therefore, model users have to be very careful to identify any differences that may exist between the reference and the target populations as well as any changes in the evolution of this target population during growth (26).

From a nutritional perspective, animal variation is much larger than the variation in feed intake and protein deposition potential as represented in actual factorial methods and models (1, 53). The actual principles used in the factorial methods to estimate nutrient requirements or to formulate low-protein diets are based on the assumptions that for many nutrients, in particular for AA, (1) digestibility is constant and is only a feed attribute [e.g., 74% for lysine in corn; NRC (1)], (2) observed (i.e., SID) AA utilization efficiency is constant for production [e.g., 72% for lysine deposition in body protein; (15)] across animals and ages (some variation is considered in the NRC 2012 model), (3) body protein amino acid composition is constant across animals and ages [e.g., 7% for lysine; (89)] and AA are needed and retained according to an ideal protein profile (1, 53, 54). However, these assumptions do not always hold true. Indeed, as indicated earlier in this document, feed ingredient AA digestibility is affected by the composition of the diet [e.g., fiber content (80, 90)], feed processing (91) and animal factors such as feed intake (78), and body weight (91). Factors affecting nutrient digestibility should be taken into account to formulate low-protein diets. In addition to this, the efficiency with which animals use the available nutrients may not be constant. For instance, the efficiency of use of the absorbed AA for protein deposition is affected by many factors in pigs, and production conditions may be one of the most important ones. Thus, in growing pigs fed below lysine requirements, the estimated SID lysine efficiency ranged from 73 to 94% (58) and from 83 to 100% (92). Similar figures were observed for threonine, where the estimated efficiency ranged from 54 to 84% (58). Amino acid supply also affects the AA composition of body proteins, and different body proteins are affected differently by the AA supply. Indeed, the splanchnic tissues are less affected than carcass muscles by AA supply, and different muscles respond differently to dietary AA supply (58, 77, 93–95). Some proteins (collagen, albumin, C-reactive protein) are also more affected than others (58). The use of constant digestibility values, AA efficiency and AA composition of body protein and animal products in the estimation of AA provisions and requirements can lead to biased estimations that can limit animal performance when trying to minimize excess nutrients supply.

The concept of the ideal protein refers to a protein with an AA profile that exactly meets the animal's requirement, and in this context all the AA acids are equally limiting (54, 77, 96). There are important implications to this concept. First, the animal response is driven by the first limiting AA, independently of the others. Second, the animal response is proportional to the available limiting AA until another AA becomes limiting or the maximal response is reached. Third, excess AA does not limit maximal response. And finally, there is no interaction between AAs. In an optimal setting, all the animals will respond similarly to a given supply of AA. However, the ideal protein concept explains a small portion of the observed variation in the animals' responses. That is, for any given level of AA supply, there is a large variation in animal responses, often larger than the variation across AA supply levels (58, 97–99). Remus et al. (77) also noted that, for growing pigs, optimal performances were obtained at different threonine/lysine ratios when pigs were fed in conventional or precision feeding systems. In both feeding systems, however, the between-animal variation was high, thus confirming that the ideal protein profile explains a relatively small proportion of the observed animal response variation. It is possible that the between-animal variation in terms of AA digestibility, the efficiency of use of available dietary AA, and AA body protein composition may be responsible for part of the unexplained animals' response variation in AA supply.

Furthermore, the utilization of the ideal protein concept is limited when a quadratic response is observed (100) or when deficiencies or excess AA affect other AA responses (AA interactions). For example, valine supplementation decreased ADG when using a diet marginal in tryptophan, whereas it increased ADG when using a tryptophan-sufficient diet (101). Valine deficiency or branched-chain AA imbalance in the diet reduced feed intake and growth performance in another trial (102). Amino acids are much more than building blocks for production. They are also essential substrates for the synthesis of many molecules (e.g., glutathione, carnitine, carnosine, etc.) crucial to the animal metabolism and they have a crucial role in neurological regulations, gene expression, and small intestine growth (103, 104). Some AA are essential to the immune system (i.e., sulfur AA) to maintain the integrity of the gut barrier (i.e., threonine), and their supply should be reviewed in pigs under poor sanitary conditions (55, 105). Functional AA are those involved in the regulation of key pathways associated with the improvement of health, growth, reproduction lactation and reproduction (106). These AA have been linked to possible metabolic disease prevention and treatment, and might have great influence on intestinal health (106, 107). Pigs in poor sanitary conditions have different AA and energy requirements than those in better conditions (108, 109). Health challenges result in shifts of AA that could be used for protein deposition being used for maintenance functions related to the immune system (108, 110, 111). As consequence non-ruminants decrease growth performance (110, 111), and this loss in efficiency using feed for growth results in increased environmental impact (112). Cadéro et al. (113) simulated 96 scenarios using a LCA model that takes into account the variability among pigs aiming to simulate the impact o health status and feeding practices on economic and environmental traits. They concluded that impaired health has a major impact on the carbon foot print, and improving practices that increase the health status also help to improve economic results. The authors point out that feeding pigs with diets that closely meet their requirements (e.g., individual precision feeding) help to improve the economic results of health impaired populations. Additionally, daily feeding groups or individually feeding pigs improved the economic and environmental performance independent of the health conditions of the herd. It is possible that the changes in functional AA concentration might help pigs overcome the sanitary challenge, especially in precision feeding systems.

## Future Perspectives

Formulating feeds with low-protein diets and feeding pigs individually or in groups with daily tailored diets can have a major impact on N excretion and overall livestock sustainability. Indeed, the ingested nutrients that are not retained by the animal or in animal products are excreted and contribute to increasing the production cost and to reducing the sustainability of the farm. Reducing the supply of AA as happens in low- and very-low-protein diets for conventional and precision feeding production systems requires integration in the estimation of AA requirements not only of their role in production (i.e., meat, milk, etc.) but also other essential metabolic functions. It also requires ensuring that other functional nutrients (e.g., fermentable carbohydrates, probiotics, etc.) are supplied to maximize the integrity of the intestinal morphology and microbiota, immune system, etc. We need to better understand AA digestion and metabolic use to quantify the animal needs and their response to AA supply in interaction with the animal microbiota and production environments.

The formulation of very-low-protein diets and the implementation of precision feeding techniques rely on the utilization of sound nutritional concepts and comprehensive biological models developed to precisely estimate individual real-time nutrient requirements and animal responses. Combining knowledge- and data-driven models will further enhance our ability to use real-time farm data, opening up new opportunities that will enhance farm profitability, nutrient efficiency, and the sustainability of the overall animal production system. With the development of advanced computer and communication technologies and high-speed data-collection sensors, it is possible today to obtain numerous measurements at the animal, feed, building, and other farm levels. Besides the availability of these new technologies and data gathering, knowledge remains the most limiting factor to precisely providing each animal or a group of animals with the amount of nutrients it needs to produce at the desired level. Understanding the metabolic processes responsible for the observed variation between individual animals in their ability to use dietary nutrients is challenging for nutritionists and modelers, but is required to further improve the efficiency of livestock production systems.

## Author Contributions

AR formulated the diets, ran the simulations, prepared tables and figures, and drafted part of the manuscript. CP reviewed the simulations and calculations provided in this document and wrote the first draft. CP, AR, and IA reviewed and edited the document. All authors read and approved the final version of the manuscript.

## Funding

The authors thank Agriculture and Agri-Food Canada and our industry partners—Elanco North America, Metex Novistago, and Adisseo France for supporting our work.

## Conflict of Interest

The authors declare that the research was conducted in the absence of any commercial or financial relationships that could be construed as a potential conflict of interest.

## Publisher's Note

All claims expressed in this article are solely those of the authors and do not necessarily represent those of their affiliated organizations, or those of the publisher, the editors and the reviewers. Any product that may be evaluated in this article, or claim that may be made by its manufacturer, is not guaranteed or endorsed by the publisher.
